# High-throughput mass spectrometry and bioinformatics analysis of breast cancer proteomic data

**DOI:** 10.1016/j.dib.2019.104125

**Published:** 2019-06-10

**Authors:** Talita Helen Bombardelli Gomig, Iglenir João Cavalli, Ricardo Lehtonen Rodrigues de Souza, Aline Castro Rodrigues Lucena, Michel Batista, Kelly Cavalcanti Machado, Fabricio Klerynton Marchini, Fabio Albuquerque Marchi, Rubens Silveira Lima, Cícero de Andrade Urban, Luciane Regina Cavalli, Enilze Maria de Souza Fonseca Ribeiro

**Affiliations:** a Genetics Department, Federal University of Parana, Curitiba, Brazil; b Functional Genomics Laboratory, Carlos Chagas Institute, Fiocruz, Curitiba, Parana, Brazil; c Mass Spectrometry Facility – RPT02H, Carlos Chagas Institute, Fiocruz, Curitiba, Parana, Brazil; d International Research Center (CIPE) – A.C. Camargo Cancer Center, São Paulo, SP, Brazil; e Breast Disease Center, Hospital Nossa Senhora das Graças, Curitiba, Brazil; f Research Institute Pele Pequeno Principe, Curitiba, Brazil; g Lombardi Comprehensive Cancer Center, Georgetown University, USA

**Keywords:** Breast cancer, Contralateral non-tumor breast tissue, LC-ESI-MS/MS, Bioinformatics

## Abstract

Data present here describe a comparative proteomic analysis among the malignant [primary breast tumor (PT) and axillary metastatic lymph nodes (LN)], and the non-tumor [contralateral (NCT) and adjacent (ANT)] breast tissues. Protein identification and quantification were performed through label-free mass spectrometry using a nano-liquid chromatography coupled to an electrospray ionization–mass spectrometry (nLC-ESI-MS/MS). The mass spectrometry proteomic data have been deposited to the ProteomeXchange Consortium via PRIDE partner repository with the dataset identifier PXD012431. A total of 462 differentially expressed proteins was identified among these tissues and was analyzed in six groups' comparisons (named NCTxANT, PTxNCT, PTxANT, LNxNCT, LNxANT and PTxLN). Proteins at 1.5 log2 fold change were submitted to the Ingenuity^®^ Pathway Analysis (IPA) software version 2.3 (QIAGEN Inc.) to identify biological pathways, disease and function annotation, and interaction networks related to cancer biology. The detailed data present here provides information about the proteome alterations and their role on breast tumorigenesis. This information can lead to novel biological insights on cancer research. For further interpretation of these data, please see our research article ‘Quantitative label-free mass spectrometry using contralateral and adjacent breast tissues reveal differentially expressed proteins and their predicted impacts on pathways and cellular functions in breast cancer’ [2].

**Table:** Specifications table

Subject area	Biology
More specific subject area	Cancer proteomics
Type of data	Table, figures
How data was acquired	Nanoliquid chromatography coupled to the nanoelectrospray mass spectrometry (LC-ESI-MS/MS)
Data format	Raw and analyzed data
Experimental factors	Samples of tumor and non-tumor tissues from breast cancer patients were collected in the same surgery procedure and stored in RNA later solution.
Experimental features	Protein extracts were isolated from tissue samples and analyzed in the LC-ESI-MS/MS using the label-free quantification (LFQ) method to obtain the protein expression levels of each condition. Statistical tests revealed the differentially expressed proteins among the tissues. These proteins were submitted to Ingenuity^®^ Pathway Analysis (IPA) software.
Data source location	Hospital Nossa Senhora das Graças, Curitiba, Paraná, Brazil.
Data accessibility	The mass spectrometry proteomics data have been deposited to the ProteomeXchange Consortium via the PRIDE partner repository with the dataset identifier PXD012431. IPA's analyses are in this article.
Related research article	T.H.B. Gomig, I.J. Cavalli, R.L.R. Souza, A.C.R. Lucena, M. Batista, K.C. Machado, F.K. Marchini, F.A. Marchi, R.S. Lima, C.A. Urban, L.R. Cavalli, E.M.S.F. Ribeiro. Quantitative label-free mass spectrometry using contralateral and adjacent breast tissues reveal differentially expressed proteins and their predicted impacts on pathways and cellular functions in breast cancer, Journal of Proteomics, 199C (2019), 1–14 [2].

**Table:** 

**Value of the data** • A differential proteome between tumor and non-tumor tissues is described, highlighting the use of a valuable biological sample as control, the contralateral non-tumor breast tissue. • The non-tumor breast tissues (NCT e ANT) present high similarity in the proteome profiling. • The common alterations in the proteomes of malignant tissues (PT and LN) point out to cancer associated proteins and pathways that can be explored in tumor progression studies. • The complete lists of differential expressed proteins and their biological context are a rich source of potential targets to be investigated in further studies.

## 1 Data

**Fig. 1:**
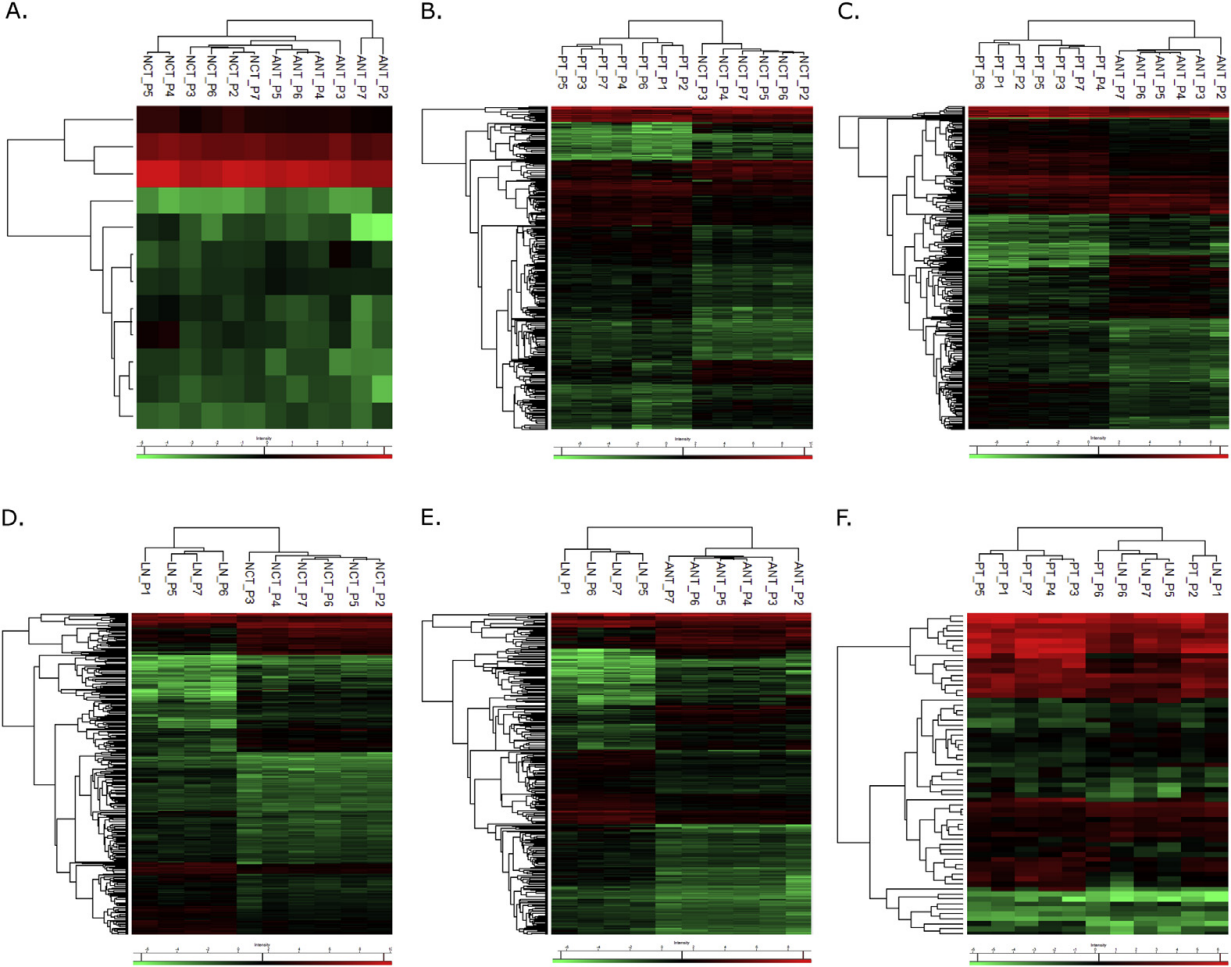
Hierarchical clustering analysis of the differentially expressed proteins for all the groups' comparisons: A. NCTxANT; B. PTxNCT; C. PTxANT; D. LNxNCT; E. LNxANT; F. TPxLN. NCT - non tumor contralateral breast tissue, ANT - non tumor adjacent breast tissue, PT - primary breast tissue, LN - axillary metastatic lymph node; P1-P7 indicate the patients.

The differential proteomic profiling of the breast cancer-related tissues was obtained using a high throughput mass spectrometry platform and appropriate statistical methods. A total of 462 identified proteins presented significant differences in the protein expression among these tissues (Supplementary File S1). Six different comparisons were performed: contralateral non-tumor breast tissue *versus* adjacent non-tumor breast tissue (NCTxANT); primary breast tumor *versus* contralateral non-tumor breast tissue (PTxNCT); primary breast tumor *versus* adjacent non-tumor breast tissue (PTxANT); axillary metastatic lymph node *versus* contralateral non-tumor breast tissue (LNxNCT); axillary metastatic lymph node *versus* adjacent non-tumor breast tissue (LNxANT); and primary breast tumor *versus* axillary metastatic lymph node (PTxLN). The differentially expressed proteins of each group’ comparison were distinctly grouped by hierarchical cluster analysis using the Perseus software version 1.5.6.0 (Fig. 1). Proteins at 1.5 log2 fold change were analyzed with IPA's tools to identify significant canonical pathways, biological functions, diseases and interaction networks for each group’ comparison (Supplementary File S2). A detailed data interpretation is available on [2].

## 2 Experimental design, material and methods

### 2.1 Protein extraction and digestion

Tissue samples were collected during the surgical procedure at Hospital Nossa Senhora das Graças at Curitiba, Parana, Brazil, and stored in RNA later solution. The samples were prepared as described in [2], according to a protocol adapted from Ostasiewicz and coworkers [3] and Tyanova and coworkers [4]. Briefly, the samples were lysed in 4% SDS, 0.1 M Tris-HCl pH 7.6 and 0.1 M DTT and homogenized in TissueLyser II sample disruptor (Qiagen Corp. MD, USA), followed by heating to 95 °C for 5 minutes. Samples were submitted to ultrasonic bath, centrifuged to remove cellular debris and processed by filter-aided sample preparation (FASP) method [5]. Proteins were briefly separated in a 1D-PAGE 10% (v/v) acrylamide gel, reduced with 10 mM DTT, alkylated with 50 mM iodacetamide and digested for 18 h with 12.5 ng/μl trypsin at 37 °C. The resulting peptides were processed to LC-ESI-MS/MS.

### 2.2 LC-ESI-MS/MS

Tryptic peptides were separated by online EASY-nLC 1000 chromatograph (Thermo Scientific) and analyzed in the LTQ Orbitrap XL ETD (Thermo Scientific). The runs were performed in triplicate for each sample. Full MS was acquired in the Orbitrap analyzer and the MS2 analysis in the ion trap analyzer, using the CID fragmentation in a DDA mode. The acquired data were analyzed in the MaxQuant software version 1.5.8.3 [6] through the Andromeda search engine [7] and the human UniProt protein database (UniProtKB [8] 24 May 2017, 70,939 entries). Raw data have been deposited to the ProteomeXchange Consortium via PRIDE [1] partner repository with the dataset identifier PXD012431. The parameters of LC-ESI-MS/MS and MaxQuant analysis are further detailed in the research article [2].

### 2.3 Data analysis

The “proteinGroups.txt” file generated by MaxQuant software was processed and analyzed in Perseus v. 1.5.6.0 [9]. Distinct tissue samples were categorized in their respective groups, including PT, LN, NCT and ANT tissues. The LFQ intensity values (that represent the protein expression levels) were log2-transformed and only proteins quantified in at least 70% of samples for each tissue were used for further analysis. Normalization was performed by width adjustment previously to the imputation of the missing values (downshift = 1.8 and width = 0.3) [10], [11]. This processed data were exported to the R platform and analyzed in RStudio version 3.4.2 (http://www.R-project.org), using in-house scripts containing the Bartlett's test, ANOVA and Duncan's test, all at significance level of 5%. Proteins that presented homogeneous variances (accessed by Bartlett's test) were submitted to ANOVA's test at p < 0.05 and FDR of 0.05. The resulting differentially expressed proteins were analyzed to identify significant differences in the mean values among the samples' pairs (Duncan's test), providing lists of the differential proteome for the six groups' comparisons (NCTxANT, PTxNCT, PTxANT, LNxNCT, LNxANT and PTxLN). Euclidean distances were used for hierarchical cluster analyses performed with the differentially expressed proteins for each group’ comparison. The 1.5 fold change cutoff was applied into the log2 data.

### 2.4 Ingenuity Pathway Analysis

Proteins at 1.5 log2 fold change of each comparative group were separately analyzed in the IPA software version 2.3 (QIAGEN Inc.) [12]. The NCTxANT group comparison was not included considering that no protein was observed at this cutoff. The gene symbols of the differentially expressed proteins and their fold change values were uploaded in IPA. The Core Analysis was performed under the following parameters: the expression fold change was set as the type of Core Analysis; direct and indirect relationships were consider to generate the networks; the prediction of these networks included the endogenous chemicals, 35 molecules per network and a total of 25 networks enabled per analysis; the confidence considers only relationships based on experimentally observed data; only the human species as well as all tissues and cell lines were set in this analysis. The cutoff values applied to all datasets included fold change ≥1.5 for up-regulated and ≤-1.5 for down-regulated proteins. Adjusted p values (Benjamini-Hochberg, FDR) of <0.05 were considered significant. Based on the IPA's analysis, significant canonical pathways, biological functions and diseases, and interaction networks were algorithmically generated, including z-score values for predict the activation status of these processes.

## Funding

This study was financed by the Coordenação de Aperfeiçoamento de Pessoal de Nível Superior - Brasil (CAPES) - Finance Code 001 and the CNPq/Araucaria Research Foundation of Parana State (PRONEX/2012).
